# Health related quality of life in patients with community-acquired pneumococcal pneumonia in France

**DOI:** 10.1186/s12955-018-0854-6

**Published:** 2018-02-02

**Authors:** Luiz Flavio Andrade, Grèce Saba, Jean-Damien Ricard, Jonathan Messika, Jacques Gaillat, Pierre Bonnin, Christian Chidiac, Hajnal-Gabriela Illes, Henri Laurichesse, Bruno Detournay, Patrick Petitpretz, Gérard de Pouvourville

**Affiliations:** 1 ESSEC Business School, Avenue Bernard Hirsch, 95021 Cergy-Pontoise Cedex, France; 2 Hôpital Louis Mourier, Assistance Publique Hôpitaux de Paris, Colombes, France; 3 INSERM, IAME, UMR 1137, University of Paris Diderot, IAME, UMR 1137, Sorbonne Paris Cité, F-75018 Paris, France; 4 CH d’Annecy Genevois, Annecy, France; 5 Hôpital de la Croix Rousse, Hospices Civils de Lyon, Lyon, France; 6 Hôpital Gabriel Montpied, CHU de Clermont-Ferrand, Clermont-Ferrand, France; 7 CEMKA-EVAL, Bourg-la-Reine, France; 8 Hôpital André Mignot, Versailles, France

**Keywords:** Pneumonia, CAP, EQ-5D, Quality of life, Tobit model

## Abstract

**Background:**

Community Acquired Pneumococcal Pneumonia is a lung infection that causes serious health problems and can lead to complications and death. The aim of this study was to observe and analyze health related quality of life after a hospital episode for patients with community acquired pneumococcal pneumonia in France.

**Methods:**

A total of 524 individuals were enrolled prospectively in the study and were followed for 12 months after hospital discharge. Presence of *streptococcus pneumoniae* was confirmed by microbiological sampling. Quality of life was reported at four different points of time with the EQ-5D-3 L health states using the French reference tariff. Complete data on all four periods was available for 269 patients.

We used descriptive and econometric analysis to assess quality of life over time during follow-up, and to identify factors that impact the utility indexes and their evolution through time. We used Tobit panel data estimators to deal with the bounded nature of utility values.

**Results:**

Average age of patients was 63 and 55% of patients were men. Negative predictors of quality of life were the severity of the initial event, history of pneumonia, smokers, age and being male. On average, quality of life improved in the first 6 months after discharge and stabilized beyond. At month 1, mean utility index was 0.53 (SD: 0.34) for men and 0.45 (SD: 0.34) for women, versus mean of 0.69 (SD: 0.33) and 0.70 (SD: 0.35) at Month 12. “Usual activities” was the dimension the most impacted by the disease episode. Utilities for men were significantly higher than for women, although male patients were more severe. Individuals over 85 years old did not improve quality of life during follow-up, and quality of life did not improve or deteriorated for 34% of patients. We found that length of hospital stay was negatively correlated with quality of life immediately after discharge.

**Conclusion:**

This study provides with evidence that quality of life after an episode of community acquired pneumococcal pneumonia improves overall until the sixth month after hospital discharge, but older patients with previous history of pneumonia may not experience health gains after the initial episode.

## Background

Community acquired pneumonia (CAP) is a lung infection that still remains associated with considerable mortality and morbidity. In Europe, the prevalence of CAP has not decreased over the past decades and is around 14 per 1000 habitants, varying widely across countries and regions. Probability of hospital admission is high for patients with CAP, putting a high strain on health resources. Because of the huge impact on heath costs and patients’ quality of life, the management of CAP is a challenge issue [1, 2]. There have being a growing number of studies focusing on patients’ perspective to assess the outcomes of health interventions. To capture health outcomes, a measure commonly used in utility analysis is the quality-adjusted life year (QALYs). The QALY metric requires a descriptive system of health states, reflecting differences in health related quality of life, and a preference-based valuation system allocating a single index to each of them [3, 4].

The EuroQol EQ-5D questionnaire [5–7] has been widely used to describe an individual’s quality of life using a descriptive system of health states on five dimensions: mobility, self-care, usual activities, pain/discomfort and anxiety/depression. Each dimension is reported with three levels of severity (EQ-5D-3 L) and more recently with five (EQ-5D-5 L). This study provides with data on health states and utility indexes after hospital discharge and over a year in individuals who were hospitalized for a Community Acquired Pneumococcal Pneumonia (CAPP). It is part of a broader study, including a cost survey in the French context. Results of the cost component have been published elsewhere [8].

The choice of focusing on CAPP, vs all hospitalized CAP, was driven by the following. First, two vaccines are available for the prevention of pneumococcal infections for adults: Pneumo 23®, a 23 valent vaccine, and Prevenar 13®, a pneumococcal polysaccharide conjugate 13-Valent vaccine. The overall goal of the broader study was thus to provide with robust data to be incorporated later in a cost-effectiveness analysis of different vaccination strategies in the French context, according to regulatory requirements for coverage and pricing. Thus, collection of health related quality of life (HRQoL) data at different points in time was required. The one-year time horizon was chosen based on the assumption that it was a reasonable time horizon for patients to recovery or stabilization of health states.

Second, the study population differs from those which were observed in the two published studies of HRQoL with EQ-5D with a one-year follow-up. In Honselman et al. [9], the study included German patients with pneumonia and/or sepsis who were admitted in intensive care units. In Mangen et al. [10] the HRQoL was assessed in patients age ≥ 65 years who were hospitalized with suspected community-acquired pneumonia, and compared to a matched population of patients without CAP living in the community. This study included a secondary comparison of HRQoL between radiological confirmed and non-confirmed community-acquired pneumonia cases. But none of the studies focused specifically on microbiologic and radiologic confirmation of *streptococcus pneumoniae*, which is the target of vaccination.

Thus, the HRQoL component of the Pneumocost study had two main immediate objectives: documenting health states and utility indexes for hospitalized CAPP patients and their evolution over time, analyzing the relationship between the characteristics of patients and patients stays with HRQoL. These research questions will lead to identification of groups of individuals in higher risk of morbidity and mortality after hospital discharge. Moreover, the ultimate goal is to use the utility data on a cost effectiveness analysis of vaccination strategies to prevent CAPP.

## Methods

### Data collection

The PNEUMOCOST study was an observational multicentre study, the primary goal of which was to evaluate the costs of hospital stays and follow-up over a 12 month period in patients with documented CAPP in France. Informed consent of patients was required, and the Consultative Committee for the Treatment of Information in Health Research (CCTIRS) and the French Data Protection Authority (CNIL) approved the study.

Overall, 41 medical units in 38 public hospitals around France included patients. A total of 524 adult patients aged 18 years and older who were hospitalized for a confirmed CAPP were enrolled prospectively. Given the patterns of missing values for the utility index, a final sample of 269 individuals was used for the descriptive and econometric analyses.

Pneumonia was confirmed at admission by X-ray and *streptococcus pneumoniae* confirmed with microbiological sampling. The two mains diagnostic tests for the bacteriological confirmation of *streptococcus pneumonia* were pneumococcal antigenuria + another test in 71.95% of cases (antigenuria alone in 42.75%) and positive blood cultures in 37.98% of cases (Blood culture alone in 13.93%) followed by a respiratory sampling, either deep expectoration in 13.74% (alone in 6.30%), or deep lung sampling either protected in 1.72% or unprotected 3.63%, a few were from pleural fluid culture (4.58) or miscellaneous in 5.92%.

Pregnant women, patients already enrolled in clinical trials, as well as individuals with prior 48 h hospital admission for another cause were excluded. We also excluded those patients unable to provide written consent or presumed to be unable to answer the questionnaires over the follow-up period. Patient data were: age, sex, diagnostic criteria for Pneumococcal CAPP, microbiological identification of S*. pneumoniae*, employment status, background and lifestyle, risk factors, vaccination profile, Charlson score [11], Port score [12].

The Charlson comorbidity index is based on the identification of the number of comorbidities of patients, using the International Classification of Diseases. The Port Score is the standard Pneumonia severity index used to predict mortality and morbidity in patients with pneumonia. It combines both comorbid conditions and clinical and biological parameters recorded during the inpatient stay. Parameters were collected during the hospital stay with an electronic Case Report Form (e-CRF). Investigators validated each questionnaire once it was completed. *Ex post* validation of completeness was counter-checked by the partner CRO. Data relative to the hospital stay itself were mode of admission, admissions in intensive care and/or other intermediate and step down care units, length of stay and billing data for the cost analysis, and were documented *ex post* using the mandatory discharge abstract system for French hospitals.

After hospital discharge, the partner CRO called patients to carry out the survey on resource use and costs, as well as quality of life during follow-up.

Health Related Quality of life (HrQoL) was measured using the three-level EQ-5D questionnaire, for which a French value set was available. The survey was carried out by phone interviews at Month 1, 3, 6 and 12 after discharge. The EQ-5D states were converted into utility values using standard French tariffs [13]. The severity of the initial CAPP episode was measured using the Port score and information on comorbidities captured with the Charlson index. EQ-5D states were not collected at admission: based on the experience of investigators, it was expected that most patients would be admitted through the A&E department in a state of respiratory failure, before being transferred to either a medical ward or to ICU, and thus be unable to answer to the questionnaire. This was confirmed subsequently.

### Statistical and econometric analysis

Descriptive statistics of the responses to the EQ-5D were used to assess trends in the different dimensions of the questionnaire over time. We then carried out multivariate analyses to identify factors associated with the utility values associated to each EQ-5D health state.

Modeling EQ-5D scores raises several issues because of the peculiar characteristics of the distribution of the utility index. The scores are limited upwards at full health (1) and − 1 for health states worse than death. The values are this distributed in a bounded interval, and there is a concentration of responses around the upper bound. Simple linear regression methods are unlike to provide robust estimations given the distribution of the utility values used as independent variable. This issue has been identified in a number of papers relative to EQ-5D modeling [14–16]. We used econometric methods recommended for censored data where the independent variable is bounded at a certain level [17]. We chose to use Tobit panel with random effect models specifying the upper limit at 1. This modeling strategy accounts not only for the censoring aspects of the data but also for the concentration of responses around perfect health. The longitudinal characteristics of the data used in this study enabled us to have more robust interpretations of the effects of the control variables. We proceeded with hierarchical models in order to capture the effects in the sequential introduction of different blocks of variables on health status.

We also carried out a descriptive statistical and econometric analysis for the group of patients who had no improvement or deterioration in their quality of life during follow up. We coded dummy variable identifying patients who had a variation equal or inferior to 0 in utility values between Month 1 and 12. We assumed that this group of patients experienced peculiar conditions after discharge and that factors associated with non-improvement may differ from the group of patients whose HrQoL improved. In this case, the dependent variable did not change over time and a simple logistic regression was used to identify the determinants of non-improvement or deterioration in HrQoL. To capture the effects of length of hospital stay on the individual health after hospital discharge, we ran Tobit models only for the values of utility at first month. The rationale behind this specification was that the effects of length of stay are more likely to be captured immediately after discharge.

A number of patients followed after discharge was not able to respond to all calls. Patterns in missing values were variable over time. To deal with missing data in our sample, we made the following choices. The descriptive analysis and the main modeling exercise were run including only individuals for whom we had full quality of life information for all periods. This full specification excluded an important share of individuals, even if some excluded patients reported health states for some periods. Overall, excluded patients were older and more severe. We used multiple imputations (MI) to calculate the EQ-5D scores for the missing values and we performed an analysis with imputed data as a sensitivity analysis, in order to verify if the missing values affected the validity of our predictions. We also included patients who died during follow-up, but only if we had complete information on their quality of life before death. For example, an individual who died at month 3 was included in the analysis if her/his utility was informed at month 1. The utility value of people that died during follow-up was coded as zero. We used STATA 14.0 to perform all the statistical analyses in this paper.

## Results

### Descriptive statistics

Table 1 describes the characteristics of the 524 patients included in the study. Mean age was 63 years (SD: 17; median: 64; min: 19; max: 96), Most of patients were men (55%); 66% were unemployed or retired. Around one fifth of patients had had a previous history of pneumonia and 55% were recorded as having a Port index of 4 or 5, which scores a severe pneumonia. The average length of hospital stay (ALOS) was 14 days (SD: 15.9; median: 10; min: 1; max: 378). As expected, 85% percent were admitted in the hospital trough the A&E department. One out of four of patients were admitted to an intensive care unit.

**Table 1:** Subject characteristics in the Pneumocost Study

Variables	Overall *N* = 524	Sample retained *N* = 269	Sample excluded *N* = 255	*P*-values for the two sample test of means and proportions
Sociodemographic				
Age				
*Mean (SE)*	63.2 (17.1)	62.0 (16.8)	64.5 (17.3)	0.101
*Range*	19–96	19–96	19–96	
Median	64	63	66	
Woman	45.0%	49.0%	40.78%	0.056
Employed	33.2%	32.7%	33.7%	0.805
BMI				
*Underweight*	10.8%	9.2%	12.5%	0.231
*Normal weight*	50.7%	49%	52.5%	0.426
*Overweight*	26.7%	29%	24.3%	0.226
*Obesity*	11.6%	12.6%	10.5%	0.464
Smoker status				
*Never smoker*	40.2%	40.5%	40.0%	0.903
*Former smoker*	29.7%	29.7%	29.8%	0.987
*Current smoker*	29.9%	29.7%	30.2%	0.909
Alcohol consumer	31.0%	35.3%	26.2%	0.025
History of pneumonia	20.0%	20%	20%	0.983
Health status				
Port Score				
*1*	7.6%	7.8%	7.4%	0.878
*2*	15.5%	20.8%	10.2%	0.000
*3*	21%	22.6%	19.2%	0.330
*4*	30.1%	30.1%	30.2%	0.983
*5*	25.5%	18.5%	32.9%	0.000
Charlson				
*1*	39.5%	45.3%	33.3%	0.004
*2*	35.8%	35.3%	36.4%	0.783
*3*	24.6%	19.3%	30.2%	0.003
Hospital stay				
Length of stay in days				
*Mean (SE)*	15.3 (22.46)	13.2 (10.2)	17.5 (30.3)	0.030
*Range*	1–378	1–87	1–378	
Median	10	10	10	
Intensive care	25.1%	21%	29%	0.049
Critical care	41.6%	22.3%	23.9%	0.660
Emergency admission rate	85%	86.5%	84.2%	0.464
Hospital mortality	*n* = 13 (2.48%)	–		
Follow-up mortality	*n* = 30 (5.73%)	*n* = 13 (4.83%)	17 (6.67%)	0.366

Two-sample test of means and proportions (T-test or Pr test) were performed between the group retained (269) and the group excluded (255)

Among people enrolled in the study, 13 died during their impatient stay and another 30 died during the follow-up period. For people who died during follow-up, full utility information was documented for 13 of them. For those who survived over a year, utility information was fully available for 256 of them, leading to a final sample size of 269 individuals (48% of the patients enrolled).

Table 1 provides with descriptive statistics for the sample that was selected for the econometric analysis as well as for those excluded because of incomplete data on health states and utility. It confirms that individuals with at least one missing value for utilities are significantly different from people that responded to all phone calls during follow-up. Patients in the analysis sample (*n* = 269) were more likely to be women (*p* = 0.056), to consume alcohol (*p* = 0.025), had a lower probability of having the worst level of the Port index (*p* < 0.001) or the worst level of the Charlson index (*p* = 0.03). Moreover, concerning hospital stays, the sample used in the econometric analysis showed shorter ALOS (*p* = 0.03) and was less likely to be admitted in the intensive care unit (*p* = 0.049).

Overall, individuals declaring better health states were younger, professionally active and men. Mean EQ-5D values declined with age. At the time of admission, the PORT severity score was significantly higher for men when compared to women (*P* < 0.002). Utility scores decreased as the severity of CAPP measured by the Port index increased. In addition, individuals having shorter inpatient stays and no history of pneumonia were more likely to report better health states after discharge. From a dynamic perspective, most of the individuals tended to declare improvement of health states over time, with a maximum value at 6 months and remaining stable thereafter. With the exception of people over 85 years, on average, quality of life was better at month 12 when compared to month 1 in all categories of patients showed in Table 2.

**Table 2:** EQ-5D scores of patients for whom we have complete information

Characteristics	*N* = 269		EQ-5D index (SD)			
Gender		1st Month	3nd Month	6th Month	12th Month	Overall (*n* = 1076)
Female	132	0.45 (0.34)	0.62 (0.34)	0.69 (0.32)	0.70 (0.35)	0.61 (0.35)
Male	137	0.53 (0.34)	0.69 (0.31)	0.69 (0.33)	0.69 (0.33)	0.65 (0.33)
Employment status						
Employed	88	0.53 (0.31)	0.79 (0.26)	0.77 (0.29)	0.82 (0.26)	0.73 (0.30)
Retired	181	0.48 (0.35)	0.60 (0.34)	0.65 (0.34)	0.63 (0.35)	0.59 (0.35)
Age (year)						
19–35	17	0.62 (0.27)	0.88 (0.26)	0.76 (0.38)	0.82 (0.26)	0.77 (0.31)
36–45	37	0.52 (0.35)	0.78 (0.26)	0.83 (0.24)	0.86 (0.27)	0.75 (0.31)
46–55	32	0.54 (0.30)	0.69 (0.33)	0.70 (0.34)	0.69 (0.33)	0.66 (0.33)
56–65	67	0.52 (0.33)	0.70 (0.29)	0.76 (0.28)	0.80 (0.26)	0.70 (0.31)
66–75	49	0.48 (0.37)	0.65 (0.31)	0.68 (0.33)	0.66 (0.34)	0.62 (0.35)
76–85	49	0.41 (0.35)	0.49 (0.35)	0.55 (0.32)	0.53 (0.38)	0.50 (0.35)
86–95	18	0.42 (0.35)	0.44 (0.35)	0.46 (0.33)	0.39 (0.27)	0.43 (0.32)
PSI/PORT						
1	21	0.55 (0.38)	0.82 (0.25)	0.82 (0.25)	0.83 (0.29)	0.76 (0.31)
2	56	0.53 (0.31)	0.76 (0.29)	0.82 (0.26)	0.80 (0.30)	0.73 (0.31)
3	61	0.58 (0.33)	0.66 (0.34)	0.66 (0.35)	0.69 (0.32)	0.65 (0.33)
4	81	0.46 (0.33)	0.61 (0.33)	0.67 (0.31)	0.68 (0.32)	0.60 (0.33)
5	50	0.38 (0.36)	0.55 (0.32)	0.57 (0.35)	0.55 (0.39)	0.51 (0.36)
History of pneumonia						
No	215	0.49 (0.35)	0.68 (0.32)	0.71 (0.32)	0.70 (0.33)	0.65 (0.34)
Yes	54	0.51 (0.31)	0.57 (0.35)	0.62 (0.35)	0.65 (0.34)	0.59 (0.34)

(SD): Standard deviation in parenthesis

**Fig. 1:**
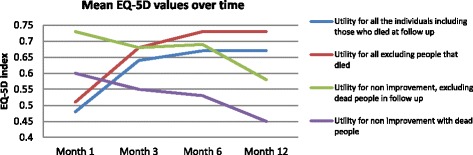
Utility values from EQ-5D over time for different groups of patients

We found a different pattern in the utility values for people who had no improvement or deterioration in health states at the end of the follow-up period. Figure 1 shows different curves for utility values over time for two groups of participants in the study. People with deterioration/no improvement in health states were on average in better health states at Month 1 after discharge than individuals who have improved through time, but then deteriorated.

In Table 3 we present an analysis of the distribution of responses according to the five dimensions and the three levels of the EQ-5D questionnaire, showing the most affected dimensions in quality of life after hospital discharge. In this table, we used all available EQ-5D information at each period, thus responder samples are not the same at each point in time. Usual activities appeared to be the health dimension for which extreme problems were most often recorded, and this, at all times of the survey (30% in the first month). Pain and discomfort was extremely high for 11% of respondents in the first month after discharge while mobility was the least affected dimension in people with CAPP. The proportion of people reporting extreme problems tended to decrease over time for all the dimensions while declarations of no problems tended to increase.

**Table 3:** Distribution in the different dimensions of the EQ-5D index over time

Problem	T1	T2	T3	T4
Mobility	*N* = 382 (%)	*N* = 347 (%)	*N* = 334 (%)	*N* = 374 (%)
No problem	134 (35.08)	196(56.48)	213 (63.77)	224 (59.89)
Moderate	227 (59.42)	149 (42.94)	119 (35.63)	144 (38.50)
Extreme	21 (5.50)	2 (0.58)	2 (0.60)	6 (1.60)
Self-care	*N* = 382 (%)	*N* = 348 (%)	*N* = 239 (%)	*N* = 372 (%)
No problem	290 (75.92)	291 (83.62)	279 (84.80)	298 (80.11)
Moderate	58 (15.18)	36 (10.34)	29 (8.81)	52 (13.98)
Extreme	34 (8.90)	21 (6.03)	21 (6.38)	22 (5.91)
Usual activities	*N* = 382 (%)	*N* = 350 (%)	*N* = 335 (%)	*N* = 374 (%)
No problem	99 (25.92)	182 (52.00)	195 (58.21)	209 (55.88)
Moderate	166 (43.46)	127 (36.29)	105 (31.34)	116 (31.02)
Extreme	117 (30.63)	41 (11.71)	35 (10.45)	49 (13.10)
Pain/ discomfort				
No problem	178 (46.72)	222 (63.43)	232 (69.25)	280 (74.87)
Moderate	159 (41.73)	107 (30.57)	80 (23.88)	80 (21.39)
Extreme	44 (11.55)	21 (6.00)	23 (6.87)	14 (3.74)
Anxiety/ depression				
No problem	214 (56.02)	218 (62.29)	243 (72.54)	270 (72.19)
Moderate	125 (32.72)	92 (26.29)	66 (19.70)	77 (20.59)
Extreme	43 (11.26)	40 (11.43)	26 (7.76)	27 (7.22)

% in parenthesis

### Multivariate estimations

Table 4 presents the results for the different modeling strategies used to assess the determinants of utility indexes after hospital discharge in patients with pneumonia.

**Table 4:** Tobit panel models with EQ-5D score (utility) as dependent variable

	Model 1	Model 2	Model 3	Model 4	Model 5	Model 6	Model 7
Age	−0.009***	− 0.007***	− 0.007***	− 0.007***	−0.006***	− 0.005**	− 0.006***
	(0.00)	(0.00)	(0.00)	(0.00)	(0.00)	(0.00)	(0.00)
Length of hospital stay	− 0.006**	− 0.004**	− 0.005**	− 0.005**	− 0.004*	− 0.004*	− 0.005**
	(0.00)	(0.00)	(0.00)	(0.00)	(0.00)	(0.00)	(0.00)
Gender (Male)	0.075	0.092*	0.093*	0.089*	0.098*	0.101**	0.090*
	(0.04)	(0.04)	(0.04)	(0.04)	(0.04)	(0.04)	(0.04)
Employment status	−0.027	−0.032	− 0.035	− 0.035	− 0.038	−0.058	− 0.068
	(0.06)	(0.05)	(0.05)	(0.05)	(0.05)	(0.05)	(0.06)
Alcohol consumption	−0.002	−0.003	− 0.003	−0.003	− 0.002	−0.001	0.000
	(0.01)	(0.00)	(0.00)	(0.00)	(0.00)	(0.00)	(0.01)
Smoker	−0.050	−0.054*	− 0.056*	−0.055*	− 0.052*	−0.057*	− 0.055*
	(0.03)	(0.02)	(0.02)	(0.02)	(0.02)	(0.02)	(0.03)
BMI	0.015	−0.001	−0.002	− 0.002	0.002	− 0.001	0.013
	(0.03)	(0.02)	(0.02)	(0.02)	(0.02)	(0.02)	(0.03)
History of pneumonia	−0.081	−0.071	− 0.070	− 0.073	− 0.071	− 0.062	− 0.067
	(0.05)	(0.05)	(0.05)	(0.05)	(0.05)	(0.05)	(0.05)
Month 1	0.000	0.000	0.000	0.000	0.000	0.000	0.000
	*Ref.*	*Ref.*	*Ref.*	*Ref.*	*Ref.*	*Ref.*	*Ref.*
Month 3	0.205***	0.203***	0.203***	0.203***	0.203***	0.203***	0.205***
	(0.02)	(0.02)	(0.02)	(0.02)	(0.02)	(0.02)	(0.02)
Month 6	0.250***	0.248***	0.248***	0.248***	0.248***	0.248***	0.250***
	(0.02)	(0.02)	(0.02)	(0.02)	(0.02)	(0.02)	(0.02)
Month 12	0.261***	0.259***	0.259***	0.259***	0.259***	0.259***	0.261***
	(0.02)	(0.02)	(0.02)	(0.02)	(0.02)	(0.02)	(0.02)
Death		−0.582***	−0.579***	− 0.583***	− 0.577***	− 0.559***	
		(0.07)	(0.07)	(0.07)	(0.07)	(0.07)	
Intensive care			0.032				
			(0.05)				
Critical care				0.041			
				(0.05)			
Port Score					−0.026		
					(0.02)		
Charlson						− 0.076*	−0.119**
						(0.03)	(0.04)
Constant	1.172***	1.094***	1.098***	1.090***	1.078***	1.034***	1.074***
sigma_u	(0.14)	(0.12)	(0.12)	(0.12)	(0.12)	(0.12)	(0.14)
Constant	0.310***	0.264***	0.264***	0.264***	0.264***	0.261***	0.303***
sigma_e	(0.02)	(0.02)	(0.02)	(0.02)	(0.02)	(0.02)	(0.02)
Constant	0.261***	0.259***	0.259***	0.259***	0.259***	0.259***	0.261***
R2	(0.01)	(0.01)	(0.01)	(0.01)	(0.01)	(0.01)	(0.01)
bic	1062.682	1005.385	1011.927	1011.601	1010.714	1007.564	1060.352
N	1112	1112	1112	1112	1112	1112	1112

Significance levels are :*p < 0.05, **p < 0.01, ***p < 0.001. Standard errors in parenthesis

Age, length of hospital stay and active smoker status were significantly correlated with lower utility values. Time dummies were introduced to capture the average trend in utility over time. Compared to the first month, utility increased significantly over the follow up period, showing a stabilization pattern between the sixth and the twelfth month. It is worth noting that it was between month 1 and month 3 that individuals experienced the highest health improvement. Men are significantly more likely to declare better quality of life than women and this association is robust in all models, except in the first one. Critical care and intensive care unit admission were not significant predictors of utility scores, and the introduction of these variables did not change the stability of the models and the significance of the others predictors, suggesting robustness in the choice of variables. The Port score was not correlated with utility, however we found that the Charlson comorbidity score was a significant predictor of lower utility values after hospital discharge in patients hospitalized with CAPP.

Employment status, body mass index and alcohol consumption had no significant impact on utility indexes.

### Determinants of deterioration/non-improvement in quality of life

From a total of 269 individuals for whom we were able to obtain quality of life data throughout the whole follow up period, 92 declared to be in equal or poorer health condition at month 12 versus month 1. We performed a statistical analysis to identify factors associated with the non-improvement in health status. First we created a two-way table describing the distribution of EQ-5D responses in patients with a decrease/stability in utility scores over the course of the study. We found that the most impacted dimensions in people with decreased/stable perceived health states were usual activities and anxiety or depression problems.

We also performed a Logit regression (Table 5) with a dichotomous dependent variable taking the value of 1 if individuals have not reported an improvement in quality of life or 0 otherwise. These specifications appeared to be better adjusted with the introduction of the variable age square as predictor. We found that participants in the study having had a previous history of pneumonia were significantly more likely to experience deterioration in health status.

**Table 5:** Logit model with dependent variable taking the value of 1 if individuals had no improvement in their health status in the course period

	Model 1	Model 2	Model 3	Model 4	Model 5
Age	−0.090	− 0.089	− 0.091	− 0.089	−0.078
	(0.05)	(0.05)	(0.05)	(0.05)	(0.05)
Age square	0.001	0.001	0.001	0.001	0.001
	(0.00)	(0.00)	(0.00)	(0.00)	(0.00)
Length of hospital stay	−0.018	− 0.017	− 0.021	− 0.021	− 0.028
	(0.02)	(0.02)	(0.02)	(0.02)	(0.02)
Gender (male)	0.475	0.471	0.455	0.459	0.402
	(0.28)	(0.28)	(0.28)	(0.28)	(0.30)
Employed	−0.494	− 0.484	− 0.514	− 0.475	− 0.456
	(0.40)	(0.40)	(0.40)	(0.40)	(0.41)
Alcohol consumption	0.014	0.014	0.014	0.012	0.017
	(0.03)	(0.03)	(0.03)	(0.03)	(0.03)
Smoker	0.206	0.210	0.202	0.198	0.202
	(0.18)	(0.18)	(0.18)	(0.18)	(0.19)
Body mass index	−0.034	−0.030	−0.036	−0.040	0.005
	(0.17)	(0.17)	(0.17)	(0.17)	(0.18)
History of pneumonia	0.642*	0.641*	0.630	0.641*	0.659
	(0.33)	(0.33)	(0.33)	(0.33)	(0.34)
Intensive care		−0.092			
		(0.35)			
Critical care			0.233		
			(0.33)		
Port score				0.081	0.076
				(0.15)	(0.15)
Dummy for death					0.000
					(.)
Constant	1.594	1.561	1.615	1.546	1.306
	(1.53)	(1.54)	(1.54)	(1.54)	(1.57)
Bic	386.882	392.409	391.985	392.176	364.662
N	269.000	269.000	269.000	269.000	256.000

Significance levels are : *p < 0.05, **p < 0.01, ***p < 0.001. Standard erros in parenthesis

### Quality of life at 1st month and length of hospital stay

We performed a specific analysis to assess the effect of length of stay on health states at Month 1 after discharge. We assumed that length of hospital stay was directly related to the clinical needs of patients, and that the impact of duration of stay on utility would be limited to the first month. We proceeded to the same modeling strategies used in the previous analysis. The only difference is that, since we modeled utility values only for the first month after hospital discharge, the models are no longer longitudinal regressions. After controlling for severity and demographic characteristics, the Tobit regression with upper limit at 1 showed that length of stay was, in all models, negatively associated (*p* < 0.001) with health states at Month 1 after discharge (Table 6).

**Table 6:** Tobit for utility values at month 1

	Model 1	Model 2	Model 3	Model 4	Model 5	Model 6
Age	−0.005**	−0.005**	−0.005*	−0.005*	−0.003	−0.005**
	(0.00)	(0.00)	(0.00)	(0.00)	(0.00)	(0.00)
Length of hospital stay	−0.009***	−0.009***	− 0.009***	−0.009***	− 0.009***	−0.009***
	(0.00)	(0.00)	(0.00)	(0.00)	(0.00)	(0.00)
Gender (male)	0.130**	0.130**	0.133**	0.133**	0.140**	0.128**
	(0.05)	(0.05)	(0.05)	(0.05)	(0.05)	(0.05)
Employed	−0.090	−0.089	−0.093	−0.093	− 0.114	−0.092
	(0.06)	(0.07)	(0.07)	(0.07)	(0.07)	(0.06)
Alcohol consumption	−0.002	−0.002	− 0.001	−0.001	− 0.000	−0.002
	(0.01)	(0.01)	(0.01)	(0.01)	(0.01)	(0.01)
Smoker	−0.029	−0.028	− 0.028	−0.028	− 0.032	−0.029
	(0.03)	(0.03)	(0.03)	(0.03)	(0.03)	(0.03)
Body mass index	0.003	0.003	0.004	0.004	0.003	0.003
	(0.03)	(0.03)	(0.03)	(0.03)	(0.03)	(0.03)
History of pneumonia	0.036	0.035	0.036	0.036	0.043	0.034
	(0.06)	(0.06)	(0.06)	(0.06)	(0.06)	(0.06)
Intensive care		−0.006	0.002	0.002	−0.005	
		(0.06)	(0.06)	(0.06)	(0.06)	
Port score			−0.011	−0.011		
			(0.03)	(0.03)		
Charlson score					−0.075	
					(0.04)	
Critical care						0.023
						(0.06)
Constant	0.942***	0.941***	0.935***	0.935***	0.879***	0.940***
	(0.15)	(0.15)	(0.15)	(0.15)	(0.15)	(0.15)
Constant	0.356***	0.356***	0.356***	0.356***	0.354***	0.356***
	(0.02)	(0.02)	(0.02)	(0.02)	(0.02)	(0.02)
R2						
BIC	308.118	313.702	319.104	319.104	316.227	313.545
N	269.000	269.000	269.000	269.000	269.000	269.000

Significance levels are: *p < 0.05, **p < 0.01, ***p < 0.001. Standard erros in parenthesis

### Sensitivity analysis with multiple imputations for the missing values of utility

We performed several multivariate analyses to test whether including individuals with incomplete HrQoL information would change the predictions of our models. We assumed that missing values were not random. A simple means comparison test showed that individuals not responding to at least one phone call in the course of the study were significantly more likely to be older (65 versus 61 years old), retired, with higher length of stay (18 days versus 12 days), and had higher Port and Charlson scores. Thus, participants with at least one missing value were in poorer health conditions than those having answered the survey in all periods of the study. The results of Tobit panel models including imputed data for missing values of utility appeared to confirm the lower quality of life of individuals with missing data. The Port score was now statistically significant in predicting utility values; in the models with multiple imputations, length of hospital stay was no longer a robust predictor of health conditions after hospital discharge. In the appendix we show analysis using the total sample of 524 individuals where the missing values were replaced for multiple imputations of the utility value (Tables 7 and 8). We can observe in Table 7 that in average, the MI reduces the mean values of utilities.

**Table 7:** EQ-5D scores including imputed values for the missing data

Characteristics	*N* = 524		EQ-5D index (SD)			
Gender		1st Month	3nd Month	6th Month	12th Month	Overall (*n* = 2096)
Female	236	0.42 (0.32)	0.58 (0.32)	0.66 (0.29)	0.65 (0.33)	0.58 (0.33)
Male	288	0.51 (0.31)	0.67 (0.29)	0.66 (0.30)	0.67 (0.31)	0.63 (0.31)
Employment status						
Employed	174	0.54 (0.27)	0.78 (0.24)	0.76 (0.26)	0.81 (0.23)	0.72 (0.27)
Retired	350	0.43 (0.33)	0.55 (0.31)	0.61 (0.30)	0.59 (0.34)	0.55 (0.33)
Age (year)						
19–35	30	0.59 (0.24)	0.87 (0.23)	0.80 (0.32)	0.86 (0.21)	0.78 (0.28)
36–45	67	0.53 (0.32)	0.78 (0.25)	0.81 (0.21)	0.89 (0.21)	0.75 (0.28)
46–55	66	0.53 (0.28)	0.70 (0.29)	0.72 (0.27)	0.73 (0.26)	0.67 (0.28)
56–65	117	0.46 (0.31)	0.65 (0.29)	0.70 (0.30)	0.70 (0.30)	0.63 (0.32)
66–75	96	0.47 (0.34)	0.60 (0.30)	0.64 (0.29)	0.63 (0.33)	0.58 (0.32)
76–85	100	0.41 (0.33)	0.52 (0.29)	0.52 (0.29)	0.53 (0.33)	0.49 (0.31)
86–95	48	0.38 (0.31)	0.40 (0.29)	0.49 (0.28)	0.37 (0.25)	0.41 (0.29)
PSI/PORT						
1	40	0.57 (0.31)	0.84 (0.20)	0.79 (0.23)	0.88 (0.22)	0.77 (0.27)
2	82	0.55 (0.29)	0.76 (0.28)	0.80 (0.25)	0.81 (0.27)	0.73 (0.29)
3	110	0.54 (0.32)	0.64 (0.31)	0.70 (0.30)	0.72 (0.27)	0.65 (0.31)
4	158	0.45 (0.29)	0.59 (0.30)	0.62 (0.30)	0.62 (0.32)	0.57 (0.31)
5	134	0.36 (0.32)	0.52 (0.29)	0.55 (0.29)	0.51 (0.33)	0.48 (0.32)
History of pneumonia						
No	419	0.47 (0.32)	0.65 (0.30)	0.68 (0.29)	0.67 (0.32)	0.61 (0.32)
Yes	105	0.47 (0.30)	0.55 (0.32)	0.59 (0.31)	0.64 (0.32)	0.56 (0.32)

(SD): Standard deviation in parenthesis

**Table 8:** Tobit panel models with imputed utility scores

	Model 1	Model 2	Model 3	Model 4	Model 5	Model 6	Model 7
Age	−0.008***	− 0.007***	− 0.008***	− 0.008***	− 0.006***	− 0.005***	− 0.005***
	(0.00)	(0.00)	(0.00)	(0.00)	(0.00)	(0.00)	(0.00)
Length of hospital stay	− 0.001	− 0.001	− 0.001	− 0.001	− 0.000	− 0.001	− 0.001
	(0.00)	(0.00)	(0.00)	(0.00)	(0.00)	(0.00)	(0.00)
Gender (Male)	0.077**	0.097***	0.077**	0.077**	0.088***	0.092***	0.106***
	(0.03)	(0.02)	(0.03)	(0.03)	(0.03)	(0.03)	(0.02)
Employed	0.020	−0.000	0.021	0.019	0.006	−0.011	− 0.023
	(0.04)	(0.03)	(0.04)	(0.04)	(0.03)	(0.03)	(0.03)
Alcohol consumption	− 0.003	− 0.004	− 0.003	−0.003	− 0.001	− 0.002	− 0.003
	(0.00)	(0.00)	(0.00)	(0.00)	(0.00)	(0.00)	(0.00)
Smoker	−0.062***	−0.063***	− 0.062***	−0.062***	− 0.058***	−0.062***	− 0.063***
	(0.02)	(0.01)	(0.02)	(0.02)	(0.02)	(0.02)	(0.01)
BMI	−0.000	−0.006	− 0.000	−0.001	0.005	0.003	−0.003
	(0.01)	(0.01)	(0.01)	(0.01)	(0.01)	(0.01)	(0.01)
History of pneumonia	−0.068*	−0.062*	− 0.068*	−0.068*	− 0.073*	−0.054	− 0.052
	(0.03)	(0.03)	(0.03)	(0.03)	(0.03)	(0.03)	(0.03)
Month 1	0.000	0.000	0.000	0.000	0.000	0.000	0.000
	(.)	(.)	(.)	(.)	(.)	(.)	(.)
Month 3	0.185***	0.185***	0.185***	0.185***	0.185***	0.185***	0.185***
	(0.02)	(0.02)	(0.02)	(0.02)	(0.02)	(0.02)	(0.02)
Month 6	0.222***	0.221***	0.222***	0.222***	0.222***	0.222***	0.221***
	(0.02)	(0.02)	(0.02)	(0.02)	(0.02)	(0.02)	(0.02)
Month 12	0.235***	0.235***	0.235***	0.235***	0.235***	0.235***	0.234***
	(0.02)	(0.02)	(0.02)	(0.02)	(0.02)	(0.02)	(0.02)
Dummy for dead		−0.383***					−0.359***
		(0.04)					(0.04)
Intensive care			−0.002				
			(0.03)				
Critical care				0.011			
				(0.03)			
Port score					−0.051***		
					(0.01)		
Charlson score						−0.105***	−0.077***
						(0.02)	(0.02)
Constant	1.035***	1.025***	1.035***	1.034***	1.019***	0.932***	0.950***
	(0.08)	(0.08)	(0.08)	(0.08)	(0.08)	(0.08)	(0.08)
Constant	0.239***	0.214***	0.239***	0.239***	0.234***	0.232***	0.210***
	(0.01)	(0.01)	(0.01)	(0.01)	(0.01)	(0.01)	(0.01)
Constant	0.247***	0.246***	0.247***	0.247***	0.247***	0.247***	0.246***
	(0.00)	(0.00)	(0.00)	(0.00)	(0.00)	(0.00)	(0.00)
BIC	1532.362	1456.856	1540.007	1539.868	1522.869	1517.090	1450.392
N	2096	2096	2096	2096	2096	2096	2096

Significance levels are: *p < 0.05, **p < 0.01, ***p < 0.001. Standard erros in parenthesis

## Discussion

To our knowledge, this is the first study assessing quality of life in patients with microbiological confirmed pneumococcal CAP using panel data analysis. The peculiarity of this study lies on the accurate identification of *Streptococcus pneumoniæ* on the observed population of individuals hospitalized with pneumonia. The availability of utility values in different time points after discharge enabled us to produce more accurate inferences on causal effects between the patient’s characteristics and his/her health related quality of life. Pneumonia remains a serious and life threatening condition. Assessing quality of life over time in patients with CAPP could help to target vulnerable groups and to develop strategies in post-discharge follow-up that could prevent morbidity and mortality.

Indeed, several factors were associated with the quality of life in patients with confirmed CAPP. Not surprisingly, the elderly were more likely to experience worse recovery from pneumonia and decreasing quality of life [18]. The time dynamic analysis of the EQ-5D scores indicated that the age group of 85 years old and over were more likely to experience a deterioration in quality of life. This suggests that several factors associated with CAPP in the elderly, such as previous hospital admissions and comorbidities, have an important impact on the ability of older people to improve and maintain satisfactory health conditions after hospital discharge. Thus, this age group should deserve special attention in the post-discharge period.

Second, our study also highlighted the health consequences of smoking in patients with CAPP. We observed that, except in model 1, perception of good health in active smokers was lower than that of patients having never smoked.

Third, our results showed a negative impact of previous medical history of pneumonia on HRQoL after discharge. The mechanism underlying the association between past respiratory disease and actual quality of life may be that lung abnormalities persist over time, making affected individuals more susceptible to new infections, and more likely to perceive their quality of life as worse in case of a subsequent respiratory disorder [19]. For this reason, this vulnerable population should be monitored to prevent the occurrence of complications after hospital discharge.

Utility values were not significantly associated with the pneumonia severity index of the initial episode. However, the Charlson comorbidity index appeared to be negatively correlated with quality of life, suggesting that individual appraisals of health after discharge may be more related to the global level of comorbidities than to pneumonia itself. This result gives some insight on the factors associated with non-improvement in quality of life during follow-up. We observed that a non-negligible number of patients declared to be in worse health in the last month of survey compared to the first month, and the only significant factor associated with this result was a history of pneumonia. Several studies have demonstrated that respiratory diseases such as bronchitis, asthma and acute pneumonia can cause other health problems such as cardiovascular diseases. This increases short term morbidity and mortality, particularly in patients that have already been admitted in hospital with pneumonia [20–22]. It is highly possible that deterioration in health here was due to other conditions than pneumonia. We also found that individuals who did not improve during follow-up declared a better quality of life at the first month than those who improved in the one-year period after discharge. This suggests that the deterioration of the quality of life of this subgroup is related to another concurrent health problem and not to the initial episode. Since this subgroup presented with a higher comorbidity background, their recovery may have been more difficult.

A complementary finding was that men tend to declare better health conditions than women after hospital discharge. By contrast, the Port and the Charlson index at the moment of admission were higher for men, indicating more comorbidities and more severe pneumonia. Because we did not have baseline values for the EQ-5D index, we were not able to compare the quality of life of men and women with subjective and objective health measures at the same time and to draw conclusions as to what extent stated quality of life was affected by gender perceptions. An extensive literature has shown that health perceptions are in part socially constructed and that the environment and risk factors can play an essential role in the individual perception of health [23–26].

Comparison with published results with an equivalent duration of follow-up was limited, because of differences in study design and patients’ profiles. Honselman et al. [9] provided QoL VAS values at 1 year after discharge, using EQ-5D 3 L for 217 patients who had been admitted in ICU for pneumonia and sepsis in one German hospital. Thus, this cohort is different from the our study cohort in terms of disease status (CAPP versus unspecified pneumonia and sepsis), age (63 years versus 71), severity (all hospitalized patients versus ICU patients) and measure of QoL index (utility based evaluation versus VAS). Not accounting for such differences, a naive comparison of results confirms the higher severity of patients in the German study. In our study, mean utility value at 1 year for survivors was 0.73 versus 0.5 (VAS score 50) in the German study. The design of the study from Mangen et al. [10] is closer to ours. Within a clinical trial aiming at measuring the impact of vaccination for pneumococcal infections in the Dutch population age 65 and over, 562 patients hospitalized with a suspicion of community acquired pneumonia (CAP) were followed during 1 year, and QoL was compared to a non-diseased group of 1123 patients with adequate matching. Because of the clinical trial design, it was possible to collect QoL at the time of vaccination, before the onset of the disease, at admission, 2 weeks after discharge, and at Months 1, 6 and 12. QoL was measured using the EQ-5D-3 L and SF 36, and valued using the Dutch utility values as well as VAS. Patients in the suspected CAP group were older than in our study (76 versus 63) with a higher percentage of males (71% versus 55%). A naïve comparison of utility values at Months 1, 6 and 12 show similar levels of utility at 6 and 12 months, but a lower level for French patients at Month 1 (0. 51 versus 0.72). Thus, the absolute variation between Month 1 and Month 12 was 0.02 for the Dutch cohort versus 0.22 for the French cohort. As in our study, the most impacted dimensions were mobility and impact on usual activity, but Dutch patients report a higher impact on pain and discomfort. Finally, the Dutch study found no differences between patients with radiologically and non-radiologically confirmed CAP. The French cohort targeted a sub-group of the first, since the target was patients with pneumonia with *streptococcus pneumoniae.* Thus, the comparison may suggest that the difference between patients with suspected CAP and confirmed pneumococcal infection in terms of quality of life is to be seen mainly on the first 30 days after discharge.

Main limitations of the results are the following. First, data collection through phone interviews led to a high rate of non-responders at different times. To deal with this issue, we chose to focus on the reference modeling exercise with patients for whom we had full information over 12 months. Patients excluded were found to be older and more severe, which led to an overestimation of estimated values, but did not change substantially the directions of results in the multivariate analysis. The inpatient mortality rate is low in our study, compared to other published study. There has been a selection bias in the inclusion of patients. Since 84.5% were admitted through the emergency room before being transferred to another ward, eligible patients were transferred directly from ER to ICUs and lost for inclusion, since it was impossible for an unknown percentage of very severe patients to collect informed consent at admission and to record initial clinical observations Moreover, patients were lost to follow-up and thus their vital status was unknown. For the same reason, investigators could not collect QoL data at admission, and inpatient QoL was not recorded. Data at discharge, or shortly after discharge was not recorded either, to minimize both investigator and patient load. Further research on the relationship between variations in health utility measures and medical expenditure could be an interesting subject for future analyses, and thus contribute to a better understanding of medical determinants of quality of life in patients with acute conditions such as pneumonia.

## Conclusion

This study provides robust estimations of QoL and utility data for patients hospitalized for an acute episode of pneumococcal CAPP. Our results suggest that the impact of an acute episode of CAPP on QoL will last 6 months for most patients, but that a small number of patients, especially the eldest, may not recover fully from the initial episode. Since our analysis was focused on patients with full utility data, the 6 months to recovery applies particularly to patients with lower severity presentations. Not surprisingly, age, severity of the initial episode, previous history of pneumonia, history of smoking and active smoking are major determinants of QoL in CAPP patients and should deserve special attention after discharge.

Since we collected data on patient and disease characteristics, our data can be used to document cost-effectiveness studies related to the prevention or treatment of pneumonia, while adjusting for different patient profiles. The study provides data both on EQ-5D-3 L health states using on utility values computed with French tariffs and provides evidence on the responsiveness of the EQ-5D questionnaire in conditions such as pneumonia. The health state data could also be valued using other published national tariffs.

## Abbreviations

A&E: Accident and emergency
CAP: Community Acquired Pneumonia
CAPP: Community Acquired Pneumococcal Pneumonia
CCTIRS: Consultative Committee for the Treatment of Information in Health Research
CNIL: French Data Protection Authority
CRO: Contract Research Organisation
e-CRF: Electronic Case Report Form
EQ-5D: EuroQol 5-Dimension
EQ-5D-3 L: EuroQol 5-Dimension − 3 Level
EQ-5D-5 L: EuroQol 5-Dimension − 5 Level
HRQoL: Health related quality of life
ICU: Intensive care units
MI: Multiple imputations
QALY: Quality adjusted life years
VAS: Visual Analogue Scale
